# A Case of Ganglionopathy Presenting with Hyperkinetic Movements

**DOI:** 10.5334/tohm.1110

**Published:** 2026-02-11

**Authors:** Sanuri Gunawardena, Umar Shuaib, Junaid Siddiqui

**Affiliations:** 1 Cleveland Clinic Neurological Institute, US; 2 Cleveland Clinic Center for Neurological Restoration, US

**Keywords:** ganglionopathy, sensory ataxia, pseudoathetosis, Sjogren’s disease

## Abstract

**Background:**

Sensory ganglionopathy is a rare, often underrecognized neurological manifestation of Sjogren’s disease, presenting with non–length-dependent sensory symptoms that may mimic other neuropathies.

**Case Report:**

We report a case of Sjogren’s-related sensory ganglionopathy presenting as severe sensory ataxia and pseudoathetosis. A 73-year-old man developed involuntary movements, sensory loss, and gait ataxia. Exam showed absent proprioception, areflexia, and worsening ataxia with eye closure. MRI revealed degenerative spine disease; EMG showed demyelinating and axonal polyneuropathy. CSF protein was elevated and Sjogren’s antibodies were positive.

**Discussion:**

Progressive sensory ataxia despite immunotherapy confirmed Sjogren’s-associated sensory ganglionopathy emphasizing the importance of early recognition.

## Introduction

The objective of this case study is to highlight the diagnostic challenges created by a rare neurological manifestation of Sjogren’s disease. Neurological involvement in Sjogren’s can affect both the central and peripheral nervous systems, and when present, it may precede glandular symptoms or the diagnosis of the underlying autoimmune condition. Sensory ganglionopathy is an uncommon and often underrecognized complication of Sjogren’s disease, characterized by a distinct clinical presentation that differs from length-dependent peripheral neuropathies. Diagnosis is challenging due to its insidious onset, non-length–dependent sensory symptoms, and overlap with other neurological or structural conditions. This case contributes to the growing recognition of sensory ganglionopathy as a distinct neurological manifestation of Sjogren’s disease. By delineating its characteristic non-length-dependent sensory loss and proprioceptive deficits, it reinforces the need for heightened clinical suspicion and tailored diagnostic evaluation in patients with atypical sensory ataxia.

The intent of this case study is to raise awareness of sensory ganglionopathy as a rare yet clinically significant neurological manifestation of Sjogren’s disease. Due to the rarity of this condition, randomized controlled trials are lacking, and treatment is largely based on case reports and small series. The diagnostic gap contributes to delays in appropriate treatment and misdiagnosis as conditions such as chronic inflammatory demyelinating polyneuropathy (CIDP) or functional movement disorders. By detailing the characteristic clinical and electrophysiologic features of Sjogren’s-related sensory ganglionopathy, this case highlights the need for enhanced diagnostic precision and comprehensive sensory examination in patients with atypical ataxia and pseudoathetosis.

## Case Description

A 73-year-old male was referred to the Cleveland Clinic Center for Neurological Restoration for involuntary movements, lower extremity weakness, and generalized pain. His surgical history included C3-C5 laminectomy and carpal tunnel release, and medical history included hypothyroidism, arthritis, and cataracts. He described his legs would “fly” out sometimes or would suddenly give out, and he had to “focus in order to bring his legs back to a good position.” He also experienced generalized pain upon standing that improved after a few steps. His electrophysiologist reported dyskinesias along with REM behavior disorder type symptoms.

The movement disorder examination was significant for choreiform movements, dystonic posturing in his right lower extremity and right upper extremity distally, and moderate to severe axial and extremity ataxia. It was subsequently noted that these symptoms worsened significantly when his eyes were closed. In addition, there was 4- to 4+ weakness of all extremities proximally and distally. He had severely diminished sensation to all modalities, predominantly in the distal distribution with almost absent proprioception of his fingers, wrists, toes, and ankles. He also had absent lower extremity deep tendon reflexes. During gait exam using his cane, the patient had an unsteady station, dystonic right lower extremity and right hand, and impaired dorsiflexion of his left foot while ambulating (Video 1).

**Video 1:** The patient from the discussed case presenting for office visit. With eyes closed, note pseudoathetosis with limited proprioception, as right-hand drifts toward face. Next note finger to nose testing with eyes open and then eyes closed, revealing visibly more pseudoataxia with eyes closed. During gait exam using cane, patient with unsteady station, dystonic right lower extremity and right hand, and impaired dorsiflexion of left foot while ambulating.

MRI lumbar spine revealed advanced multilevel degenerative changes, multilevel spinal canal stenosis, and advanced multilevel facet arthrosis with grade one anterolisthesis of L4 on L5. EMG revealed left C6 and C7 motor radiculopathies, left L5 and S1 motor radiculopathies, and severe peripheral polyneuropathy with both segmental demyelination and axonal loss. Nerve conduction studies in both arms found minimally prolonged distal motor latencies of the left ulnar nerve at the wrist. Right median nerve palmar and distal sensory latencies were delayed. Motor conduction velocities of both peroneal nerves and the right tibial nerve were decreased. The F-wave latencies of both peroneal and tibial nerves were delayed. Needle EMG was normal in both proximal and distal muscles, tested in all four extremities. His CSF testing revealed elevated protein at 83 but otherwise was unremarkable. His serum studies were notable for a positive Sjogren’s antibody (anti-SSA).

After his first visit on 7/6/22, he was initially treated with IVIG every four weeks and Plaquenil for presumed CIDP associated with Sjogren’s disease. On his first follow-up on 10/30/23 he was still using a walker and had trouble gripping it with his hands. His right hemibody was overall worsened and areflexia was now diffuse. He was given a short course of prednisone and referred to neuromuscular clinic for what was initially thought to be refractory CIDP related to Sjogren’s disease after approximately fifteen months of treatment, however, patient chose to follow-up locally for which records are not available. On further evaluation of his exam findings and progressive worsening despite treatment, his clinical picture is more suggestive of Sjogren’s sensory ganglionopathy.

## Discussion

Sensory ganglionopathy (sensory neuronopathy) is a rare but disabling neurological manifestation of Sjogren’s disease, occurring in fewer than 5% of affected patients. Notably, neurological symptoms may precede the diagnosis of Sjogren’s by several years, making early recognition crucial. In a 2023 systematic review, Liampas et al. reported that neuropathic symptoms preceded or coincided with the diagnosis of primary Sjogren’s syndrome in a 2:1 ratio, underscoring the importance of neurologic manifestations as an initial clue to the underlying systemic disease [1]. Patients often present with asymmetric paresthesias, initially affecting the digits of the hands and feet, and gradually progressing over months or years to involve the upper extremities, trunk, and face. Among Sjogren’s-related peripheral neuropathies, distal axonal polyneuropathy is most common (~80%), followed by sensory ganglionopathy and peripheral or cranial mononeuropathies [1].

In our case, the patient presented with severe pseudoathetosis and sensory ataxia prior to a diagnosis of Sjogren’s disease, and his profound proprioceptive loss prompted further investigation ultimately uncovering the diagnosis of a sensory ganglionopathy due to Sjogren’s disease. This highlights the importance of a thorough sensory examination, especially in cases where movement abnormalities may mimic primary motor disorders, or when presented with symptoms that worsen upon eye closure. The non-length-dependent, sensory-predominant pattern with minimal true weakness, and relative preservation of needle EMG findings, in this patient contrasts with the symmetric, length-dependent sensory loss and prominent motor involvement typical of CIDP, emphasizing the value of accurate clinical localization. Furthermore, spine pathology—which may coexist or appear on imaging—can be a misleading distractor in reaching the correct diagnosis.

Due to the rarity of this condition, randomized controlled trials are lacking, and treatment is largely based on case reports and small series. Early observational data suggested a potential role for immunomodulatory therapy; notably, Molina et al. (1996), reported symptomatic improvement in a small series of patients with Sjogren’s associated sensory ganglionopathy treated with intravenous immunoglobulin (IVIG), highlighting the possibility for short-term benefit in selected cases [2]. However, subsequent studies demonstrated more variable outcomes. In a cohort of thirteen patients reported by Pereira et al. (2016), multiple immunomodulatory agents were used (Table 1), yet functional outcomes slightly worsened over a median three-year follow-up (modified Rankin Scale from 2.15 to 2.38), with the most favorable responses observed in patients treated with corticosteroids combined with mycophenolate mofetil, while IVIG showed limited benefit [3].

**Table 1:** Summary of Observational Studies Discussing Treatments for Sjogren’s Associated Sensory Ganglionopathy.


STUDY	N	THERAPIES USED	OUTCOME

Molina et al., 1996	Small series	IVIG	Short term symptomatic improvement

Pereira et al., 2016	13	Corticosteroids, mycophenolate mofetil, hydroxychloroquine, IVIG, cyclophosphamide	Slight mRS worsening over 3 years

Khan et al., 2019	1	Corticosteroids, cyclophosphamide, rituximab → MMF	Sustained functional recovery


A more promising result was reported by Khan et al. (2019), who described a 45-year-old man with primary Sjogren’s and severe sensory ganglionopathy unresponsive to steroids and IVIG. He was subsequently treated with a regimen of pulsed methylprednisolone (1 g for 3 days), cyclophosphamide (6 months), and rituximab (two infusions of 1 g), followed by a tapering course of oral glucocorticoids. After initial clinical improvement, he transitioned to maintenance therapy with mycophenolate mofetil (1 g twice daily). At three-month follow-up and after a period of rehabilitation, he was able to return to work full-time and remained clinically stable one year after initiating therapy [4]. This case illustrates the potential utility of B-cell–targeted therapy (rituximab) in refractory cases and underscores the need to consider sensory ganglionopathy early in the differential diagnosis of sensory ataxia or unusual movement disorders, particularly in patients with or at risk for Sjogren’s disease.
